# Breast Cancer Awareness, Screening Practices, and Perceived Barriers Among Female Undergraduate Students: An Institution‐Based Cross‐Sectional Study

**DOI:** 10.1002/cnr2.70187

**Published:** 2025-03-18

**Authors:** Md. Mayin Uddin Hasan, Mohammad Injamul Hoq, Rijuana Ireen Tanju, Md. Jakaria, Mohammed Abu Sayeed

**Affiliations:** ^1^ Department of Pharmacy International Islamic University Chittagong Chittagong Bangladesh; ^2^ Drug Insides and Disease Epidemiology (DIDE) Chittagong Bangladesh; ^3^ Department of Public Health University of Creative Technology Chittagong Chittagong Bangladesh; ^4^ School of Health Sciences and Purdue Institute for Integrative Neurosciences Purdue University Indiana USA

**Keywords:** awareness, breast cancer, breast screening, BSE, CBE, perceived barrier, practice

## Abstract

**Background:**

Breast cancer is a significant worldwide public health issue that poses a substantial threat to the lives of countless people around the world. Increasing awareness and implementing screening for breast cancer are two of the crucial strategies for decreasing the burden of disease. The study assessed awareness, practice, and perceived barriers to breast cancer screening among university students.

**Method:**

An institution‐based cross‐sectional study was conducted from June 2023 to December 2023 at the International Islamic University Chittagong (IIUC), Bangladesh, where 387 students were randomly selected for this study.

**Result:**

Among 387 participants, the majority (25.06%) were from the faculty of business administration, whereas 23.51% were from the faculty of science and engineering. Most (80.41%) of respondents had sufficient awareness of breast cancer and its screening. While they were aware of breast cancer screening, majority (67.7%) did not practice it. Students of law faculty were about three times [AOR: 0.31 (95% CI: 0.118–0.828)] and students of business administration faculty were about five times [AOR: 0.21 (95% CI: 0.087–0.532)] less likely to be aware of breast cancer and Breast Self Examination (BSE) than faculty of science and engineering. Also, living in dormitory students were more than two times [AOR: 0.42 (95% CI: 0.189–0.940)] less likely to be aware of breast cancer and screening of breast than those living with family. The majority (52.2% to 79.33%) of the students did not correctly identify different warning signs of breast cancer. Perceptions of having “no signs & symptoms,” not having “sufficient organization working on screening,” and “lack of awareness program” were found to be leading barriers to breast screening among students.

**Conclusion:**

Though better awareness about breast cancer and its screening was found among the students, low practices were observed in screening. Students of the science and engineering faculty demonstrated a sufficient level of breast cancer awareness than students from other faculties, and students with good screening practice habits of breast cancer were sufficiently aware of breast cancer than poorly practicing students. Still, warning signs of breast cancer are unfamiliar to female students among university students. Frequent awareness campaigns are essential to train the students at regular intervals.

## 1 Introduction

Worldwide, breast cancer is one of the significant public health issues that kill several people [1]. According to the research, the global incidence of breast cancer in 2020 was approximately 2.3 million cases, resulting in 685 000 deaths [2, 3]. Developed countries, particularly the United States, showed a high incidence of breast cancer while developing nations have lower incidence rates but higher death rates [4, 5, 6]. Poor healthcare infrastructure and varied methods of breast cancer diagnosis contribute to the fact that developing nations account for over 60% of all breast cancer fatalities [7, 8].

An estimated 13 000 women in Bangladesh are diagnosed with breast cancer each year, with over 7000 losing their lives to the disease, according to statistics from the International Agency for Research on Cancer (IARC) [9, 10]. The projected incidence of breast cancer in Bangladesh is 22.5 per 100 000 females of all ages. Among Bangladeshi women aged 15–44 years, the disease is most common (19.3 per 100 000), the highest among all cancers [10, 11, 12]. In the last 5 years, the incidence of breast cancer in Bangladesh has been reported as 32.8%, with breast cancer accounting for 69% of all female deaths caused by cancer in the country [13, 14].

There is an increase in the incidence of different cancers, including breast cancer, among young women in developing countries like Bangladesh [15]. Public health policies regarding breast cancer are fundamental in low‐ and middle‐income countries. It is necessary to create and implement affordable methods for screening and treatment, reduce risk factors, and construct an adequate cancer infrastructure [16, 17]. Policies like the implementation of awareness campaigns or interventions are effective in increasing awareness about breast cancer and enhancing breast cancer screening rates among women [18, 19]. A strong association was found between awareness level and early detection of breast cancer by screening from previous studies [20, 21]. Moreover, in developing countries, breast cancer cases are diagnosed at an advanced stage rather than at an early stage. Awareness of early warning signs and screening techniques like breast self‐examination (BSE) and clinical breast examination (CBE) helps detect breast cancer at an early stage [22]. In developed countries, healthcare workers train the general population regarding the need for screening to attend screening sessions. Hence, the number of screenings of breast cancer participants has been increasing over the years [23, 24]. However, developing countries still lack such strategies to reduce the breast cancer burden. Several studies have been targeting university students to estimate their awareness and practicing behaviors for breast cancer [25, 26, 27]. However, a few studies have been conducted in Bangladesh focusing only on university students.

We focused specifically on university students as this group is getting the tertiary level of education in this region. We hypothesize that the awareness level and screening practice for breast cancer are comparatively higher than those of average community people. The study aims to discover breast cancer awareness and practice among tertiary‐level students. The findings could be essential in universities' interventions. If the students possess adequate awareness and regular practicing habits of breast cancer, the university can engage them as potential health educators to make them aware of community people [28]. On the other hand, the lack of sufficient awareness among students encourages the university authorities to take initiatives to enhance awareness campaigns on university premises. This institution‐based study also evaluated the factors associated with breast cancer screening practices among university students in Bangladesh.

## 2 Methodology

### 2.1 Methodology

A descriptive cross‐sectional study was conducted among female students at the International Islamic University of Chittagong (IIUC), Bangladesh, from June 2023 to December 2023.

The selection process was based on the fact that this university is among the most densely populated in the southeastern region of Bangladesh, bringing students from multiple districts. It gathers students from various areas, including rural and urban students.

A multistage sampling technique was used to select the study participants. First, eight departments were selected randomly from the 13 departments among five faculties using simple random sampling. All educational years (first to fourth) of the selected departments were included, and a proportional number of study participants was allocated based on the total number of students in each chosen department and faculty. A systematic technique was applied to select the final respondents, using the class register as the sampling frame. The inclusion criteria for participants were regular students of a specific department, aged 18 or older, and willing to participate. Exclusion criteria encompassed students under 18, individuals with any physically or mentally disabled condition, and those unwilling to participate.

### 2.2 Data Collection Tools and Procedure

A structured questionnaire was designed using previously published studies [29, 30, 31] to get information from the participants. The questionnaire was closed‐ended and compiled from studies that were already published and had the following sections: sociodemographic characteristics of individuals (nine questions), questions on breast cancer awareness and practice about breast examination (eight questions), awareness of breast cancer warning signs and symptoms (nine questions), and the perceived barriers to engaging in breast cancer examination (six questions) [29, 30, 31].

The investigators and female volunteers responsible for data collection were trained to familiarize themselves with the research questionnaire. They also attended several seminars on breast cancer awareness. The questionnaire was translated into the local language for the participants to understand it better. The researchers and volunteers asked the questions formulated for the participants who consented to participate in the study. The volunteers and investigators were present during the face‐to‐face interviews of participants to explain and clarify if there were any difficulties in understanding or doubts arising on research questions. 15–20 min was taken as the survey duration from each participant.

### 2.3 Sample Size Calculation

The study sample was calculated using the formula *n* = *Z* ^2^ *pq*/*d* ^2^, where *n* = required sample size, *p* = expected (0.5) proportion of the study population with awareness and practice of breast cancer screening, *q* = 1 − p, and *d* = level of precision at 5%. *n* = 1.96^2^ × 0.5 × 0.5/(0.05)^2^ = 384. By rounding up, 400 participants were approached for study participation, and 387 (96.75%) agreed to participate.

### 2.4 Scoring System

According to previous research, participants were asked a question to evaluate their awareness of breast cancer and breast screening. In this question, an affirmative response indicated a good understanding of breast cancer and screening. The participants were asked a set of *eight* questions. A score of ‘1’ was given for each correct answer, while a score of ‘0’ was given for incorrect answers or responses of “do not know.” Additionally, participants were asked *four* questions on breast screening practice. Correct responses were assigned a score of 1, and incorrect responses or responses indicating uncertainty were given 0. Regarding the classified category, a score of ≤ 50% indicates insufficient awareness (4 or less) and poor practice (2 or less). In contrast, a score of > 50% indicates sufficient awareness (more than 4) and good practice (more than 2) [31, 32].

### 2.5 Data Analysis

All analyses used the STATA (StataCorp LLC, College Station) statistical package version 17. Descriptive statistics were used to describe the sociodemographic characteristics. The association between categorical variables was determined using the chi‐square test. Univariable and multivariable logistic regression was applied to identify significant predictors of breast cancer awareness and breast screening practice. The selection of independent variables for multivariable logistic regression was based on the univariate analysis having a *p* < 0.05. We also considered an odds ratio (OR) and a 95% confidence interval (CI) to analyze the degree to which dependent and independent variables are associated. Moreover, participants' thoughts on the signs of breast cancer and perceived barriers to screening among university students were displayed descriptively.

### 2.6 Ethics Issues

The Institutional Review Board (IRB) of the Department of Pharmacy, International Islamic University Chittagong, Kumira, Chittagong‐4318, Bangladesh Ref: IRB/ph‐36(1)/ASA/23 approved the study. Confidentiality of participants' responses was maintained, and the participants provided written consent to be included in the study.

## 3 Results

### 3.1 Demographic Status

Out of 387 female undergraduate university students who participated in the study, the mean (±SD) age of participants was 21.61(1.37). The majority of students enrolled in the faculties of business administration (25.06%) and science and engineering (23.51%), with a notable proportion in their first year of undergraduate studies (28.68%). 9.3% of the individuals were married, 11.11% lived in hostels, and 24.03% came from rural regions before admission. The majority of students are Muslim (97.93%), whereas the other religion is Hindu (2.07%). Most students (34.63%) had a monthly family income of 40 001–60 000 Bangladesh taka (BDT) (Table 1).

**TABLE 1:** Sociodemographic characteristics of study participants.

Demographic status	Frequency (*n* = 387)	Percentage (%)
Age (mean ± SD)	21.61(±1.37)	
Category of faculty		
Science and engineering	91	23.5
Social science	65	16.8
Humanities	60	15.5
Law	74	19.1
Business administration	97	25.1
Enrolled education year		
First year	111	28.7
Second year	98	25.3
Third year	90	23.3
Fourth year	88	22.7
Marital status		
Single	351	90.7
Married	36	9.3
Types of family		
Nuclear	291	75.2
Joint	96	24.8
Current residence		
Living with family	344	88.9
Living in hostel	43	11.1
Residence before admission		
Urban	294	76.0
Rural	93	24.0
Religion		
Muslim	379	97.9
Hindu	8	2.1
Family income		
< 20 000BDT	34	8.8
20 000–40 000BDT	92	23.8
40 001–60 000BDT	134	34.6
More than 60 000BDT	127	32.8
Family history of breast cancer		
No	336	86.8
Yes	51	13.2

Abbreviation: BDT = Bangladesh taka.

### 3.2 Assessment of Awareness

Among 387 female students, 98.45% had heard about breast cancer, but only 9.3% participated in any breast cancer‐related campaigns or programs. While 82.43% knew about BSE, but the majority (67.7%) did not know how to perform this examination. Among all students, 13.18% reported having a family history of breast cancer **(**Table 2). Examining the percentages of sufficient and insufficient awareness across diverse demographic factors. Notably, business administration and law faculties have higher percentages of sufficient awareness at 39.47% and 22.37%, respectively. Among education years, first year students showed the highest proportion of sufficient awareness at 34.21%, surpassing their peers. Urban residents show a notable percentage of sufficient awareness at 71.05%, compared to 28.95% for rural residents. However, no clear distinctions are observed based on marital status, family structure, or religious affiliation. However, respondents with good screening practices exhibit higher percentages of sufficient awareness at 2.63%, emphasizing the potential connection between awareness and proactive health behaviors (Table 3).

**TABLE 2:** Awareness of breast cancer and breast screening.

Awareness about breast cancer and breast screening		Response	Freq. (*n* = 387)	Percentage (%)
1	Have you ever known about breast cancer?	No	6	1.6
		Yes	381	98.5
2	Is it a severe disease?	No	6	1.6
		Yes	364	94.1
		Don't know	17	4.4
3	Is it a preventable disease?	No	24	6.2
		Yes	289	74.7
		Don't know	74	19.1
4	Only females can be affected by breast cancer.	No	92	23.8
		Yes	209	54.0
		Don't know	86	22.2
5	Breast can be transmitted from one person to one person?	No	304	78.6
		Yes	17	4.4
		Don't know	66	17.1
6	Have you ever joined any breast cancer awareness program?	No	351	90.7
		Yes	36	9.3
7	Do you know about what breast self‐examination is?	No	68	17.6
		Yes	319	82.4
8	Have you ever heard about the clinical screening program for breasts?	No	68	17.6
		Yes	319	82.4

**TABLE 3:** Association between sociodemographic characteristics and breast cancer and screening awareness.

Demographic status	Insufficient *n* = 76(%)	Sufficient *n* = 311(%)	Total *n* = 387 (%)	Chi‐square	*p*
Faculty					
Science and engineering	8 (10.53)	83 (26.69)	91 (23.51)	15.77	0.003^*^
Social science	11 (14.47)	54 (17.36)	65 (16.8)		
Humanities	10 (13.16)	50 (16.08)	60 (15.5)		
Law	17 (22.37)	57 (18.33)	74 (19.12)		
Business administration	30 (39.47)	67 (21.54)	97 (25.06)		
Total	76 (100)	311 (100)	387 (100)		
Enrolled education year					
First year	26 (34.21)	85 (27.33)	111 (28.68)	5.56	0.135
Second year	21 (27.63)	77 (24.76)	98 (25.32)		
Third year	10 (13.16)	80 (25.72)	90 (23.26)		
Fourth year	19 (25)	69 (22.19)	88 (22.74)		
Total	76 (100)	311 (100)	387 (100)		
Marital status					
Single	66 (86.84)	285 (91.64)	351 (90.7)	1.66	0.197
Married	10 (13.16)	26 (8.36)	36 (9.3)		
Total	76 (100)	311 (100)	387 (100)		
Types of family					
Nuclear	56 (73.68)	235 (75.56)	291 (75.19)	0.11	0.734
Joint	20 (26.32)	76 (24.44)	96 (24.81)		
Total	76 (100)	311 (100)	387 (100)		
Current residence					
Living with family	63 (82.89)	281 (90.35)	344 (88.89)	3.44	0.064
Living in hostel	13 (17.11)	30 (9.65)	43 (11.11)		
Total	76(100)	311(100)	387 (100)		
Residence before admit					
Urban	54 (71.05)	240 (77.17)	294 (75.97)	1.25	0.263
Rural	22 (28.95)	71 (22.83)	93 (24.03)		
Total	76 (100)	311 (100)	387 (100)		
Religion					
Muslim	75 (98.68)	304 (97.75)	379 (97.93)	0.26	0.608
Hindu	1 (1.32)	7 (2.25)	8 (2.07)		
Total	76 (100)	311 (100)	387 (100)		
Monthly family income (BDT)					
1000–20 00	9 (11.84)	25 (8.04)	34 (8.79)	3.11	0.374
20 001–40 000	13 (17.11)	79 (25.40)	92 (23.77)		
40 001–60 000	29 (38.16)	105 (33.76)	134 (34.63)		
More than 60 000	25 (32.89)	102 (32.8)	127 (32.82)		
Total	76 (100)	311 (100)	387 (100)		
Family history of breast cancer					
No	68 (89.47)	268 (86.17)	336 (86.82)	0.58	0.446
Yes	8 (10.53)	43 (13.83)	51 (13.18)		
Total	76 (100)	311 (100)	387 (100)		
Breast cancer screening status					
Poor practice	74 (97.37)	265 (85.21)	339 (87.6)	8.31	0.004*
Good practice	2 (2.63)	46 (14.79)	48 (12.4)		
Total	76 (100)	311 (100)	387 (100)		

Abbreviation: BDT = Bangladesh taka.

^*^ Statistically significant ~*p* = < 0.05.

### 3.3 Associated Factors With Awareness of Breast Cancer and Its Screening

Univariate logistic regression analysis found that faculty, enrolled education year, and breast cancer screening status were profoundly associated with awareness about breast cancer and its screening. However, multivariable logistic regression demonstrated that after accounting for these factors, students of law faculty were three times [AOR: 0.31 (95% CI: 0.118–0.828)] and students of business administration faculty were five times [AOR: 0.21 (95% CI: 0.087–0.532)] less likely to be aware of breast cancer and its screening than faculty of science and Engineering. Also, living in a hostel, students were more than two times [AOR: 0.42 (95% CI: 0.189–0.940)] less likely to be aware of breast cancer and its screening than those living with Family. However, students with a 20 001–40 000 family income were three times [AOR: 2.97 (95% CI: 1.038–8.515)] more likely to be aware of breast cancer and its screening than those in the lower income group. Furthermore, good screening practicing students were five times [AOR: 5.0146 (95% CI: 1.133–22.181)] more aware than poor practicing students (Table 4).

**TABLE 4:** Logistic regression of the level of awareness and sociodemographic characteristics.

Variable	Category of awareness			Univariate		Multivariate	
	Insufficient (%) *n* = 76	Sufficient (%) *n* = 311	Total (%) *n* = 387	OR (95% CI)	*p*	OR 95% CI)	*p*
Faculty							
Science and engineering	8 (10.53)	83 (26.69)	91 (23.51)	1		1	
Social science	11 (14.47)	54 (17.36)	65 (16.8)	0.234 (0.178–1.251)	0.132	0.526 (0.187–1.483)	0.225
Humanities	10 (13.16)	50 (16.08)	60 (15.5)	0.481 (0.178–1.301)	0.15	0.527 (0.184–1.507)	0.232
Law	17 (22.37)	57 (18.33)	74 (19.12)	0.323 (0.130–0.799)	**0.014**	0.313 (0.118–0.828)	0.019*
Business administration	30 (39.47)	67 (21.54)	97 (25.06)	0.215 (0.092–0.500)	**0.001**	0.216 (0.087–0.532)	0.001***
Enrolled education year							
First year	26 (34.21)	85 (27.33)	111 (28.68)	0.900 (0.460–1.761)	0.759	0.625 (0.294–1.328)	0.222
Second year	21 (27.63)	77 (24.76)	98 (25.32)	1.009 (0.501–2.034)	**0.03**	0.706 (0.324–1.537)	0.381
Third year	10 (13.16)	80 (25.72)	90 (23.26)	2.202 (0.959–5.055)	1.86	1.831 (0.759–4.415)	0.178
Fourth year	19 (25)	69 (22.19)	88 (22.74)	1		1	
Marital status							
Single	66 (86.84)	285 (91.64)	351 (90.7)	1		1	
Married	10 (13.16)	26 (8.36)	36 (9.3)	0.602 (0.276–1.309)	0.201	0.435 (0.175–1.078)	0.072
Types of family							
Nuclear	56 (73.68)	235 (75.56)	291 (75.19)	1		1	
Joint	20 (26.32)	76 (24.44)	96 (24.81)	0.905 (0.510–1.604)	0.734	0.928 (0.492–1.748)	0.818
Current residence							
Living with family	63 (82.89)	281 (90.35)	344 (88.89)	1		1	
Living in hostel	13 (17.11)	30 (9.65)	43 (11.11)	0.517 (0.255–1.047)	0.067	0.422(0.189–0.940)	0.035*
Residence before admission							
Urban	54 (71.05)	240 (77.17)	294 (75.97)	1		1	
Rural	22 (28.95)	71 (22.83)	93 (24.03)	0.726 (0.413–1.273)	0.264	0.704 (0.375–1.321)	0.276
Religion							
Muslim	75 (98.68)	304 (97.75)	379(97.93)	1		1	
Hindu	1 (1.32)	7(2.25)	8(2.07)	1.726 (0.209–14.251)	0.612	1.656 (0.178–15.385)	0.657
Monthly family income (BDT)							
1000–20 000	9 (11.84)	25 (8.04)	34 (8.79)	1		1	
20 001–40 000	13 (17.11)	79 (25.40)	92 (23.77)	2.187 (0.836–5.722)	0.111	2.973 (1.038–8.515)	0.042*
40 001‐ 60 000	29 (38.16)	105 (33.76)	134 (34.63)	1.303 (0.548–3.098)	0.549	1.580 (0.619–4.030)	0.338
More than 60 000	25 (32.89)	102 (32.8)	127 (32.82)	1.46 (0.610–3.535)	0.391	1.999 (0.768–5.199)	0.156
Family history of breast cancer							
No	68 (89.47)	268 (86.17)	336 (86.82)	1		1	
Yes	8 (10.53)	43 (13.83)	51 (13.18)	1.363 (0.612–3.035)	0.447	1.240 (0.515–2.983)	0.63
Breast cancer screening status							
Poor practice	74 (97.37)	265 (85.21)	339 (87.6)	1		1	
Good practice	2 (2.63)	46 (14.79)	48 (12.4)	6.422 (1.52–27.080)	**0.011**	5.014 (1.133–22.181)	0.034*

*Note:* Bold values represent statistically significant results (*p* < 0.05). Significance levels are indicated as follows: *p < 0.05, **p < 0.01, ***p < 0.001.

Abbreviation: BDT = Bangladesh taka.

### 3.4 Participants' Awareness of Breast Cancer Warning Signs and Symptoms

**FIGURE 1:**
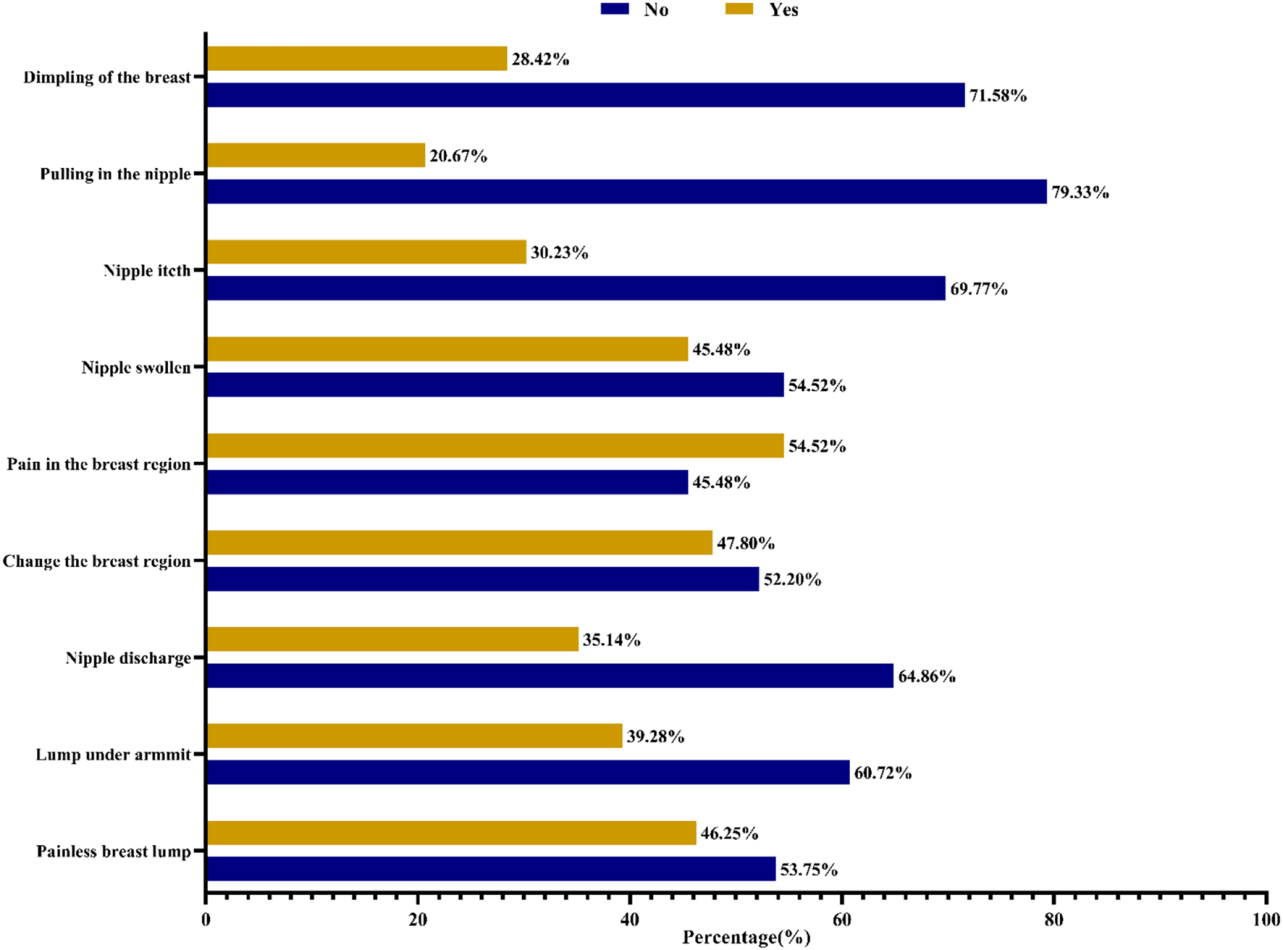
Participant's awareness of breast cancer warning signs and symptoms.

The bar diagram depicted in Figure 1 illustrates the signs and symptoms of breast cancer. Among the total (387) participants, it was reported that a painless lump (46.25%) in the breast was the notably mentioned sign of breast cancer, followed by a lump under the armpit (39.28%). The majority of the respondents did not consider nipple itch (69.77%), dimpling of the breast (71.58%), pulling the nipple (79.33%), nipple discharge (64.86%), and lumps under the armpit (60.72%) as the warning signs and symptoms of breast cancer (Figure 1).

### 3.5 Perceived Barriers Toward Breast Cancer Screening

**FIGURE 2:**
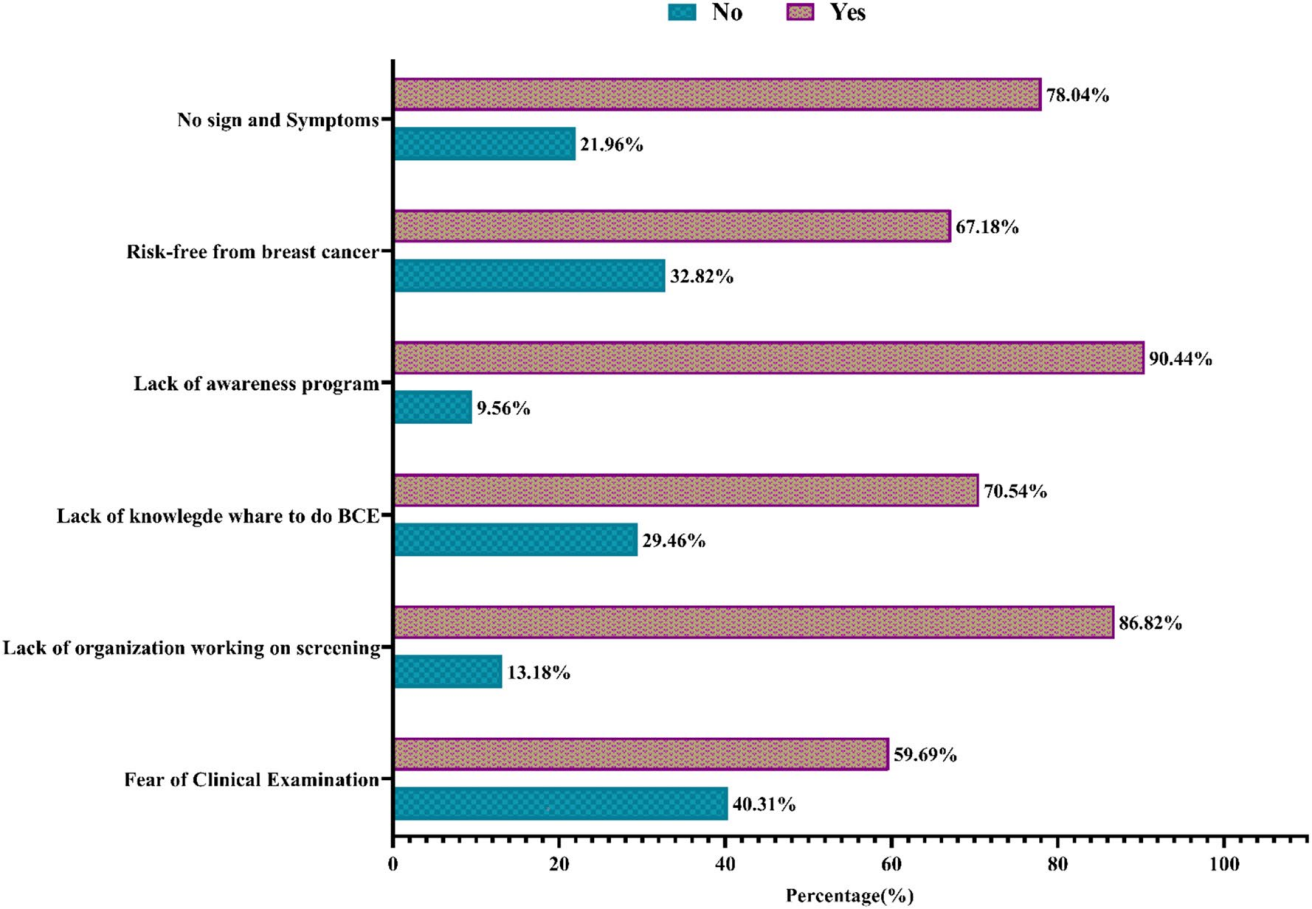
Perceived barrier of breast cancer examination.

The bar diagram depicted in Figure 2 illustrates the significant perceived barriers to breast cancer screening. As hurdles to accessing information about breast cancer screening, a total of 90.44% of respondents indicated a “lack of awareness programs,” 86.82% reported “lack of governmental and non‐governmental organizations working on breast cancer screening,” and 78.04% reported “no sign and symptoms” as the significant barriers of breast cancer screening.

On the other hand, a slightly lower portion of respondents (59.69%) reported feeling fear of clinical examination, 67.18% of respondents perceived risk‐free from breast cancer, and 70.54% of respondents reported a lack of knowledge of where to do breast clinical examination as perceived barriers to breast cancer screening (Figure 2).

## 4 Discussion

This is one of the institution‐based studies examining awareness, practice, and perceived barriers regarding breast cancer screening among Bangladeshi university students. The study's findings indicate that a significant proportion had a risk of breast cancer and had sufficient awareness regarding breast cancer and its screening, while a majority had a poor practice of breast cancer screening. Sarkar et al. found that sociodemographic characteristics such as age, education, marital status, and eating habits were important risk factors for breast cancer in Indian women [33]. In one study, only 19.4% of women routinely completed BSE, even though it is crucial for early diagnosis [34]. Compared to rural women, Indonesian urban women showed higher attitudes and actions regarding breast cancer awareness but a worse understanding of risk factors [35]. Consequently, Several meta‐analyses stated a similar pattern of better awareness of breast cancer and its screening among women [36, 37]. Despite having a good appreciation of breast cancer awareness among women, screening is remarkably limited among Pakistani women [38]. The findings from studies conducted among students in Bangladesh, Pakistan, Saudi Arabia, and Cameroon suggest the prioritization of the improvement of breast cancer screening and practice [39, 40, 41, 42]. This similar trend of having sufficient awareness and low practice could be due to similar cultural and sociodemographic characteristics across the regions.

Our research revealed that a more significant proportion of students with bachelor's degrees and higher‐income families resided with their families in urban areas, where they exhibited superior screening awareness and proficiency compared to their classmates. A recent study in Bangladesh shows that increasing awareness of the significance of screening may be crucial in encouraging women in this region to participate in screening [43]. This study also found that the rate of participation in breast cancer screening is low among students from law and business faculties and students who live in dormitories. Besides, students are more likely to engage in group discussions in hostels, like BSE, which are often considered shameful [44]. No significant difference was observed in breast cancer and its screening among students in this study. It was found that the rates of breast cancer screening were similar across different levels of education in developed countries like the United States [45].

A recent predictive analysis revealed the educational disparity. Students from law and business were three to four times less likely to perform BSE and CBE than science and engineering faculty students. Recent studies from other regions have shown similar disparities in BSE practice [39, 46]. Similarly, the knowledge and practice of breast cancer screening were significantly higher among medical students than non‐medical students [47]. A study among Saudi Arabian students showed substantially better breast screening knowledge by students studying health sciences [48]. In addition, our ongoing research also examined additional sociodemographic factors such as household income and familial history of breast cancer. The students with a monthly family income between 20 001–40 000 BDT were about three times [AOR: 2.97 (95% CI: 1.038–8.515)] more likely to be aware of breast cancer and its screening. However, no significant association was seen between a family history of breast cancer and awareness of breast cancer and breast screening. Another study objective was to evaluate the perceived barrier to screening and the level of awareness and adherence to screening practices. The study findings show that a majority of respondents have obstacles in screening due to fear of clinical examination and the absence of observable signs and symptoms in the breast. Our study showed that the absence of awareness programs was a significant barrier to obtaining information regarding screening. Several studies validate the findings [49, 50]. We observed that a substantial majority, almost 80%, of our participants were aware of breast cancer and breast screening; they hadn't actively engaged in the practice, a more significant percentage than the following findings [51]. Similar studies show that women are well aware of breast cancer and screening procedures, yet there is a significant gap in practice. In Jordan, 76% of women were aware of breast cancer incidence, yet mammography screening rates remained low [52]. Consequently, in India, 62.99% of people knew of breast cancer, with 78.67% aware of screening procedures, but practice levels were low [37]. Research in Nepal discovered that 64.57% of female support personnel were unaware of breast cancer and screening, with just 37% having used screening procedures [53]. While 87.3% of Saudi women were aware of BSE, 90.7% practiced unsatisfactory screening [54]. Conversely, research in Bangladesh revealed that 59% of university staff who knew breast screening and genuinely practiced it [29]. Furthermore, the percentage was notably greater among students with higher levels of education. Our findings indicate that there is a strong correlation between awareness of breast cancer and the actual practice of breast screening. These connections have been shown in many similar studies [55, 56].

Although our study is among the few initiatives to investigate the awareness and execution of breast screening among university students, it did have several limitations. The primary constraint of the study lies in the selected demographic group. We acknowledge that the optimal demographic would consist of mature women with little or no educational experience. University students were chosen for their convenience and to gain an early understanding of breast screening among younger, educated women.

One of the study's strengths is the execution of random sampling to choose samples from various departments inside the institution. A significant sample size was collected from university faculty, potentially reflecting the characteristics of the entire student population. The application of a cross‐sectional study design does not provide conclusive data on the causal relationship between risk factors and breast cancer screening. The findings of this research may be used to construct focused interventions for health education and health promotion initiatives that encourage breast cancer screening in developing countries like Bangladesh.

## 5 Conclusion

This study found better awareness and low levels of practice of breast cancer in a university in the southeastern part of Bangladesh. The level of awareness and practice was relatively higher in their senior education year. This study reveals that science and engineering faculty students had better practicing habits. The absence of a breast cancer awareness program served as a prominent obstacle to breast cancer screening, highlighting insufficient knowledge and a lack of organizations engaged in screening among university students. Authorities should strategically organize and implement awareness initiatives to enhance students' understanding of BSE, increase breast cancer awareness, and encourage them to undergo screening. This study suggests the implementation of health education and health promotion interventions that are accepted by the community. It also recommends expanding awareness campaigns and increasing the scale of the screening program at the university.

## Author Contributions

M.U.H. conceived and designed the study; wrote, analyzed, and interpreted the data. M.I.H. conceived the study; wrote, interpreted the data, and supervised the study. R.I.T. contributed in data collection, data curation, logistic support, and wrote the manuscript. M.J. wrote and reviewed the manuscript and supervised the study. M.A.S. reviewed the manuscript and supervised the study.

## Ethics Statement

Ethical approval was taken from the IRB of the Department of Pharmacy, International Islamic University Chittagong, Kumira, Chittagong, Bangladesh, for this work.

## Conflicts of Interest

The authors declare no conflicts of interest.

## Data Availability

The corresponding author, Mohammad Injamul Hoq, assures that all authors have read and approved the final version of the manuscript; he had full access to all of the data in this study and takes complete responsibility for the data's integrity and the data analysis's accuracy.
